# Management of ongoing direct anticoagulant treatment in patients with hip fracture

**DOI:** 10.1038/s41598-021-89077-8

**Published:** 2021-05-04

**Authors:** Carlo Rostagno, Alessandro Cartei, Gianluca Polidori, Roberto Civinini, Alice Ceccofiglio, Gaia Rubbieri, Massimo Curcio, Alberto Boccaccini, Adriano Peris, Domenico Prisco

**Affiliations:** 1 Dipartimento Medicina Sperimentale e Clinica, Università di Firenze, Florence, Italy; 2 SOD medicina interna e post chirurgica AOU Careggi, Florence, Italy; 3 SOD anestesia e rianimazione AOU Careggi, Florence, Italy; 4 Dipartimento neuromuscoloscheletrico AOU Careggi, Florence, Italy; 5 Department of Experimental and Clinical Medicine, Chief of Medicina Interna e postchirurgica, University of Florence, AOU Careggi Firenze, Viale Morgagni 85, 50134 Florence, Italy

**Keywords:** Drug regulation, Fracture repair, Health policy, Therapeutics

## Abstract

Aim of the present study was to investigate the effects of ongoing treatment with DOACs on time from trauma to surgery and on in-hospital clinical outcomes (blood losses, need for transfusion, mortality) in patients with hip fracture. Moreover we evaluated the adherence to current guidelines regarding the time from last drug intake and surgery. In this observational retrospective study clinical records of patients admitted for hip fracture from January 2016 to January 2019 were reviewed. 74 patients were in treatment with DOACs at hospital admission. Demographic data, comorbidities and functional status before trauma were retrieved. As control group we evaluated 206 patients not on anticoagulants matched for age, gender, type of fracture and ASA score. Time to surgery was significantly longer in patients treated with DOACs (3.6 + 2.7 vs. 2.15 ± 1.07 days, p < 0.0001) and treatment within 48 h was 47% vs. 80% in control group (p < 0.0001). The adherence to guidelines’ suggested time from last drug intake to surgery was 46%. Neither anticipation nor delay in surgery did result in increased mortality, length of stay or complication rates with the exception of larger perioperative blood loss (Hb levels < 8 g/dl) in DOACs patients (34% vs 9% p < 0.0001). Present results suggest that time to surgery is significantly longer in DOAC patients in comparison to controls and adherence to guidelines still limited.

## Introduction

Hip fractures occur in about 18% of women and 6% of men in their ninth decade^1,2^. In the world the number of hip fractures is expected to increase from 1.26 million in 1990 to 4.5 million by the year 2050^3^. Early surgery remains the mainstay of treatment in these patients^4^. Chronic oral anticoagulation, involving at least 15% of hip fracture population, has been reported to significantly increase time to surgery and length of hospital stay^5–7^ . However, limited data exist about direct anticoagulant agents (DOACs) in this setting^8–11^. Aim of present study was to investigate the effects of ongoing treatment with DOACs on time to surgery and clinical outcomes in patients with hip fracture. Moreover we evaluated the adherence to guideline’s suggested time to surgery in relation to the last drug intake^12,13^.

## Methods

The study is part of a project of Italian Health Ministry and Regione Toscana—RF-2010-2316600—and was approved by Ethical Committee of Regione Toscana. All patients and/or their legal guardians gave written informed consent to treatment and collection of clinical data for research purposes at admission. All research was performed in accordance with relevant guidelines/regulations. Aim of this observational retrospective study was to evaluate the effects of ongoing treatment with DOACs at the moment of trauma on time to surgery and on in hospital clinical outcomes (blood losses, need for transfusion, mortality) in elderly patients with hip fracture. Moreover we evaluated the adherence to current guidelines regarding the time from last drug intake and surgery. The clinical records of patients admitted for hip fracture in the period January 2016–January 2019 to a single tertiary teaching trauma center in Italy were reviewed. Patients were treated by a multidisciplinary team according to a previously described model^14^. From the records all the patients in treatment with a DOACs (Dabigatran Etexilate, Rivaroxaban, Apixaban, Edoxaban) at hospital admission were extracted. Data from records of 206 patients referred for hip fracture but not on anticoagulants, matched for age, gender, type of fracture and ASA score were considered as control.

### Data collection

Demographic data, comorbidities, functional status before trauma and clinical outcome were recalled from clinical records. Age, sex, type and dose of DOACs, indication to treatment, time from the last drug intake, time to surgery, length of hospital stay, need for blood transfusion and postoperative complications were recorded. Laboratory data including blood cell count, PT- INR and aPTT, creatinine and troponin were recalled from electronic records. Creatinine clearance was calculated with the Cockroft-Gault formula.

### Adherence to DOACs withdrawal guidelines

We evaluated the adherence to suggested time from last intake to surgery, recalled from pharmacological reconciliation, according to EHRA guidelines and derived Perioperative Anticoagulation Use for Surgery Evaluation (PAUSE) cohort study criterions^12,13^. High risk elective surgery was allowed 48 h after the last administration for apixaban, edoxaban and rivaroxaban while in patients treated with dabigatran timing was related to renal function (expressed as creatinine clearance).

### Statistical analysis

Continuous variables were reported as mean and standard deviation. Non continuous variables were reported as frequency of distribution. Statistical analysis of continuous data was performed using non-parametric Mann–Whitney test while non continuous data were analyzed using Fisher’s exact test. A probability value of 0.05 was considered statically significant.

## Results

Seventy-four consecutive patients in treatment with a DOACs at the moment of hospitalization for hip fracture were included in the study. Indication to anticoagulation and relative distribution of treatments are reported in Table 1. More than 90% of patients were under treatment for non-valvular atrial fibrillation. Dabigatran, apixaban and rivaroxaban had similar distribution while only 3 patients were in edoxaban.

**Table 1:** Indication for antiocoagulant therapy and distribution of differente DOAC among patients.

Indication for anticoagulation	n	%
Atrial fibrillation	68	91
Venous thromboembolism	5	7
Other	1	2
**DOA**		
Dabigatran	28	38
Rivaroxaban	19	26
Apixaban	24	32
Edoxaban	3	4

Demographic and clinical characteristics of study and control group are reported in Table 2. Mean age was not statistically different (84 ± 5.4 years in DOACs patients vs 84.1 ± 8.5 years in controls). Similarly, no gender differences was found between the two groups (female sex was 68% in DOACs patients vs 76% in controls). Again there was no significant difference in type of fracture and surgical intervention between groups. The incidence of single comorbidities was not significantly different between the groups.

**Table 2:** Characteristics of DOACs patients and controls.

	DOA (74)	Controls (206)	p
Mean age	84 ± 5.4	84.1 ± 8.5	ns
Gender M/F	24/50 (68%)	49/157 (76.2%)	ns
**Type of fracture**			
Neck fracture	36	107	ns
Per trochanteric	36	93	ns
Subtrochanteric	2	6	ns
**BADL**			
≤ 4	21 (28%)	50 (25%)	ns
**Previous mobility**			
> 50 m	20	65	ns
< 50 m	29	74	ns
Home confined	25	67	ns
**ASA score**			
3	69	195	ns
4	5	11	
**Comorbidities**			
Ischemic heart disease	12 (16%)	31 (15%)	ns
Heart failure	11 (14%)	16 (8%)	
Chronic obstructive pulmonary disease	6 (8%)	27 (13%)	ns
Chronic renal failure	5 (6%)	14 (7%)	ns
Cancer	6 (8%)	20 (5%)	ns
Dementia	19 (25%)	44 (21%)	ns
Hypertension	44 (59%)	141 (68%)	
Diabetes mellitus	17 (10%)	37 (18%)	ns
Parkinson disease	4 (7.5%)	19 (5%)	ns
More than two comorbidities	39 (52%)	125 (60%)	0.036

Time to surgery was significantly longer in patients treated with DOACs (Table 3). Actually, 46% of patients were treated within 48 h in comparison to 80% of those in the control group (p < 0.0001). No significant differences were found in hospital length of stay between the groups. There were no intraoperative complications, in particular no complications related to spinal anesthesia in DOACs patients. Post-operative complications are reported in Table 4.

**Table 3:** Comparison of mortality rate and hospitalization between patients taking anticoagulants and controls.

	DOA (74)	Controls (206)	p
Time to surgery	3.6 ± 2.7	2.15 ± 1.07	< 0.0001
% treated < 48 h	47%	80%	< 0.0001
Length of hospitalization	14 ± 4.7	14.6 ± 5.6	ns
In-hospital mortality	1 (1.5%)	9 (3.4%)	ns

**Table 4:** Comparison of postoperative complications between patients taking anticoagulants and controls.

Postoperative complications	DOA (74)	Controls (206)	p
Anemia (Hb < 8.0 g/dl)	28 (37%)	26 (12%)	< 0.0001
Red blood cell transfusion	46%	41%	ns
Respiratory failure	2 (3%)	8 (3.8%)	ns
Acute renal failure	2 (3%)	7 (3.3%)	ns
Pneumonia	7 (9%)	6 (2.9%)	ns
Sepsis	2 (3%)	3 (1.4%)	ns
Wound infection	1 (1.3%)	1 (0.3%)	ns
Pulmonary embolism	0 (0.6%)	1 (0.3%)	ns
Deep vein thrombosis	4 (5.4%)	18 (8.7%)	ns
Acute myocardial infarction	2 (3%)	3 (1.4%)	ns
Acute heart failure	5 (6.5.8%)	7 (3.3%)	ns
Delirium	18 (24%)	35 (16.9%)	ns
Stroke	0 (0%)	3 (1.4%)	ns

Severe anemia, defined as post-operative Hb concentration < 8 g/dl was significantly more frequent in patients treated with DOACs than in controls although the number of patients undergoing red blood cell transfusion did not differ between groups. The hospital mortality was not different between the two groups. Finally we did not find any significant difference in bleeding related to surgery between dabigatran and direct factor Xa inhibitors (neither as post operative anemia nor as percentage of patients needing transfusions).

### Adherence to present recommendations regarding time to surgery in patients on DOACs

The adherence to EHRA guidelines and PAUSE cohort study criterions about time from the last drug intake to surgery was 46% (34/74 patients on DOACs) (Table 5). Surgery was performed earlier than suggested in 8 patients on dabigatran (7 had creatinine clearance between 30 and 50 ml/min and 1 < 30 ml/min) and in one patient in the other 3 drugs (all of them had creatinine clearance < 30 ml/min). In 2 patients on dabigatran, 6 on rivaroxaban and 5 on apixaban time to surgery was between 48 and 72 h. Ten, including the 2 on dabigatran, had a creatinine clearance > 50 ml/min. Finally 17 patients had a delay to surgery > 72 h not justified by a drug-related higher hemorrhagic risk. For 9 of them, for whom time to surgery exceeded 5 days, the delay was due to the need for clinical stabilization of concomitant comorbidities diagnosed at hospital admission (3 had pneumonia with respiratory failure, 1 urinary sepsis, 1 heart failure complicating myocardial infarction, 3 hyperkinetic delirium and 1 advanced neoplasm). In the other 8 patients delay was related to organization problems.

**Table 5:** Adherence to EHRA guidelines and PAUSE suggestions.

Creatinine clearance	Dabigatran			Rivaroxaban			Apixaban			Edoxaban		
	< 48 h	48–72 h	> 72 h	< 48 h	48–72 h	> 72 h	< 48 h	48–72 h	> 72 h	< 48 h	48–72 h	> 72 h
> 50 ml/min	**9**	2	4	**3**	4	4	**4**	4	3	**1**	0	0
30–50 ml/min	7	**4**	0	**3**	2	1	**4**	1	4	0	0	1
< 30 ml/min	1	0	**2**	1	0	**2**	1	0	**2**	1	0	0

Bold values indicate patients in whom surgery timing agreed to guidelines.

Neither anticipation nor delay in surgery did result in increased mortality or complication rates in comparison to control group.

## Discussion

Since their introduction in clinical practice DOACs progressively replaced antivitamin K for thromboembolic prophylaxis in particular in patients with non valvular atrial fibrillation^15, 16^. Ageing is related both to an increased need for non cardiac surgery^17, 18^ and to an increased incidence of atrial fibrillation. Several suggestions for withdrawal of anticoagulant treatment have been proposed for elective surgery based essentially on the hemorrhagic risk related to surgical procedure and the value of creatinine clearance^19, 20^. EHRA guidelines suggest to refer to these indication also for time-dependent surgery^12^.

Since about 15% of patients with hip fracture are in anticoagulant treatment at the moment of trauma, it may be estimated that in Italy at least 15,000 anticoagulated patients are annually admitted for hip fracture. Early surgery (usually within 48 h from trauma) is associated with a lower short and long-term mortality and to a better functional outcome^4, 21, 22^ . Perioperative hemorrhagic risk related to anticoagulation may significantly delay surgery and worsen prognosis^5–9^.

Despite the clinical relevance, few studies investigated perioperative management of DOACs in hip fracture patients. In the study by Tran et al.^8^ 27 patients in treatment with DOACs were included. Time to surgery was significantly longer in these patients (average 66.9 h) in comparison to 233 patients treated with antivitamin K (average 36.4 h) and 260 not anticoagulated patients (26.2 h).

Similar results were reported by Razvan et al.^9^. Overall anticoagulated patients had a longer time to surgery and a longer length of stay (LOS) than control group. One hundred and forty of 229 anticoagulated patients were on warfarin, 46 on rivaroxaban, 10 on dabigatran and 33 on apixaban. Time to surgery in mean has been 28 h in patients on warfarin and between 9 and 20 h longer for DOAC.

Mullins et al.^10^ evaluated whether early surgery may be safely performed in patients in treatment with DOACs. Among 125 patients who underwent hip surgery 63 were on DOACs at admission (14 Apixaban, 5 Dabigatran e 44 Rivaroxaban). The end points of the study were surgical bleeding, need for transfusion, reintervention rate and 30 day mortality. According to developed protocol, time to surgery did not differ between anticoagulated and control group (19.4 h) with 87% treated within 36 h. There was not significant difference in blood loss although the rate of transfusion was higher in DOAC patients (18 vs 10%). Reintervention rate was also higher in anticoagulated patients (5% vs 0%). All cause mortality was 2% in DOAC patients and 8% in controls. The study has several limitations. First, the authors do not report specified transfusion criteria not allowing any comparison with the literature; second, postoperative hemoglobin was measured on the first postoperative day and not at the nadir that often occurs at day 2 to 5 after the operation. Finally, no detailed information is provided about anesthesiologic technique and comorbidities. A recent investigation clearly stated that in patients in DOACs neuraxial anesthesia was associated with a significant delay to hip surgery in comparison to general anesthesia^11^. A small pilot study evaluated the safety of DOACs in elderly patients with hip fracture^23^. DOACs concentrations were measured at admission and before surgery. Time to surgery was ≥ 48 h from the last intake of drug in 7/11 patients and 5, all treated with apixaban, had still therapeutic concentrations of drugs at surgery. These results suggested a reduced drug elimination rate in elderly and may explain the significant higher fall in hemoglobin in patients on DOACs in comparison to patients on warfarin.

The results of present investigation confirm that in patients on treatment with DOACs at the time of trauma, surgery for hip fracture is delayed at least 24 h in comparison to non-anticoagulated patients. Only 48% patients were treated within 48 h in comparison to 80% of control group. However this delay is conceivable since almost all patients in our institution had hip surgery performed under neuraxial anesthesia. When we evaluated the adherence to guidelines suggesting time from last intake and surgery it was overall limited (46%). Surgery was performed earlier than suggested in 11% of patients. Most of these were subjects in dabigatran and with decreased creatinine clearance. Delay of surgery by 24 h in these patients should improve safety of neuraxial anesthesia and decrease the risk of bleeding. In theory with close adherence to guidelines, in our population 79% of patients might had surgery within 48 h from trauma. Nevertheless nine out of the 17 patients with surgery delayed > 72 h needed clinical stabilization before surgery. We did not find any significant difference in postoperative complications between patients in DOACs and control group.

Severe anemia (Hb < 8/dl) was significantly more frequent in DOACs patients. Surgery performed earlier than suggested (11% of patients) as well the persistence of therapeutic concentrations of DOACs in about 50% of patients treated within 48 h, as suggested by the paper of Viktil et al.^23^, may be responsible for increased perioperative blood loss. Although we did not have any anesthesiologic complication, therapeutic DOACs concentrations may carry an higher risk in patients undergoing neuraxial anhestesia.

## Limitations

The observational retrospective design is the main limitation of the study. Moreover the number of patients included is relatively small, although higher than in all the previous investigations, thus not allowing definite conclusions. Although indication to transfusion in our center is a fall in Hb below 8.0 g/dl, the decision to transfuse in frail patients is discretionally left to the clinician and this may account for the absence of significant differences in transfusion rate between DOACs patients and controls despite the difference in severe anemia found between groups. Anyway, the observational nature of the study offers the advantage of describing a real-world behavior.

## Conclusions

Time to surgery is significantly longer in DOACs patients in comparison to controls and adherence to guidelines still limited, less that 50%. Nevertheless, we did not find any significant difference in clinical outcomes (death, length of stay, complication rate) between anticoagulated and not anticoagulated patients with the exception of a larger perioperative blood loss in DOACs patients. Further studies evaluating DOACs concentration before surgery may allow to suggest ideal timing of surgery and proper anesthesiologicstrategy.
